# Lesions in deep gray nuclei after severe traumatic brain injury predict neurologic outcome

**DOI:** 10.1371/journal.pone.0186641

**Published:** 2017-11-02

**Authors:** Frédéric Clarençon, Éric Bardinet, Jacques Martinerie, Vincent Pelbarg, Nicolas Menjot de Champfleur, Rajiv Gupta, Eléonore Tollard, Gustavo Soto-Ares, Danielle Ibarrola, Emmanuelle Schmitt, Thomas Tourdias, Vincent Degos, Jérome Yelnik, Didier Dormont, Louis Puybasset, Damien Galanaud, for the Neuro Imaging for Coma Emergence and Recovery (NICER) consortium

**Affiliations:** 1 Department of Neuroradiology, Pitié-Salpêtrière Hospital, Paris, France; 2 Paris VI University, Pierre et Marie Curie, Paris, France; 3 Institut du Cerveau et de la Moelle épinière–ICM. CNRS UMR 7225; 4 Bioinformatics and Biostatistics Plateform, IHU-A-ICM, Brain and Spine Institute (ICM), Paris, France; 5 Department of Neuroradiology, Guy de Chauliac University Hospital, Montpellier, France; 6 Department of Neuroradiology, Massachusetts General Hospital, Boston, Massachusetts, United States of America; 7 Department of Neuroradiology, Rouen University Hospital, Rouen, France; 8 Department of Neuroradiology, Roger Salengro Hospital, Lille, France; 9 CERMEP, Pierre Wertheimer Neurological & Neurosurgical Hospital, Bron, France; 10 Department of Neuroradiology, Nancy University Hospital, Nancy, France; 11 Department of Neuroradiology, Bordeaux University Hospital, Bordeaux, France; 12 Neurosurgical Intensive Care Unit, Pitié-Salpêtrière Hospital, Paris VI University, Paris, France; 13 INSERM U679, Pitié-Salpêtrière Hospital, Paris VI University, Paris. France; University of South Florida, UNITED STATES

## Abstract

**Purpose:**

This study evaluates the correlation between injuries to deep gray matter nuclei, as quantitated by lesions in these nuclei on MR T2 Fast Spin Echo (T2 FSE) images, with 6-month neurological outcome after severe traumatic brain injury (TBI).

**Materials and methods:**

Ninety-five patients (80 males, mean age = 36.7y) with severe TBI were prospectively enrolled. All patients underwent a MR scan within the 45 days after the trauma that included a T2 FSE acquisition. A 3D deformable atlas of the deep gray matter was registered to this sequence; deep gray matter lesions (DGML) were evaluated using a semi-quantitative classification scheme. The 6-month outcome was dichotomized into unfavorable (death, vegetative or minimally conscious state) or favorable (minimal or no neurologic deficit) outcome.

**Results:**

Sixty-six percent of the patients (63/95) had both satisfactory registration of the 3D atlas on T2 FSE and available clinical follow-up. Patients without DGML had an 89% chance (P = 0.0016) of favorable outcome while those with bilateral DGML had an 80% risk of unfavorable outcome (P = 0.00008). Multivariate analysis based on DGML accurately classified patients with unfavorable neurological outcome in 90.5% of the cases.

**Conclusion:**

Lesions in deep gray matter nuclei may predict long-term outcome after severe TBI with high sensitivity and specificity.

## Introduction

Deep gray matter (DGM) consists of the bilateral thalami and several gray matter nuclei regrouped under the name basal ganglia (BG). The BG include the neostriatum (consisting of the caudate nucleus [CN], putamen, and nucleus accumbens [NA]), globus pallidus (GP) (composed of GP externa and GP interna), substantia nigra (SN) (composed of pars compacta and pars reticulata), zona incerta (ZI), red nucleus (RN) and sub-thalamic nucleus (STN).

Severe traumatic brain injuries (TBI) are usually observed secondary to high velocity head trauma [1]. Several demographic and clinical data, like age, sex (male), Glasgow coma scale (GCS) at admission or pupillary response [2, 3] may influence the neurological outcome in severe TBI. Most of the intracranial intraparenchymal traumatic lesions are located in the frontobasal area and at anterior aspect of the temporal lobes [4]. Lesions’ topography depicted on CT-scan [3], morphologic MRI [5] and multimodality MRI [6–8] has also a strong influence on the long-term clinical outcome. For instance, it has been demonstrated that thalamic, corpus callosum and brainstem traumatic lesions are associated with a poor neurological outcome [5, 9]. Traumatic lesions of the DGM are frequent in severe TBI and are found in up to 46% of cases [10]. Multiple case reports and series document lesions in DGM in comatose patients [11–13]. However, only scant data are available on the influence of DGM lesions (DGML) on the clinical outcome in patients with severe TBI.

The purpose of this work was to evaluate the correlation between the DGML’ burden depicted on T2 Fast Spin Echo (T2 FSE) images and long-term neurological prognosis in patients with severe TBI.

## Materials and methods

### Patients

The institutional review boards of the Pitié-Salpêtrière Hospital and all participating institutions approved the study. Written informed consent was obtained for all study participants (patient’s next of kin during the acute stage, and patients themselves after recovery of consciousness). The protocol was registered on December 2007 (NCT00577954).

One hundred and five consecutive patients were prospectively enrolled from May 2001 to March 2009 in 6 different University Hospital Centers. Retrospective analysis of these prospectively collected data was conducted for this study.

All patients had severe traumatic brain injury (TBI) defined as an inability to follow simple commands that could not be explained by sedation one week after severe head trauma. The inclusion criteria were: (1) adult patient between 15 and 75 year of age with severe TBI independently from the mechanism, except penetrating injury; and (2) comatose state with inability to follow simple commands that could not be explained by sedation at least 7 days, and not more than 45 days, after TBI. Exclusion criteria were: (1) moribund patients (expected survival < 24h); (2) physiological instability (e.g., due to hemodynamic instability, increased intracranial pressure, and/or rapidly deteriorating respiratory function) that would preclude MRI scanning; (3) contraindication to the MRI; (4) penetrating head injury; (5) major intraparenchymal, subdural or epidural hematoma requiring emergent surgical evacuation or decompressive craniectomy; and (6) a central nervous system condition such as stroke, brain tumor, or a neurodegenerative disease preceding TBI.

Patients’ demographics are summarized in **Table 1**.

**Table 1:** Demographic characteristics of patients involved in our study.

Characteristics	Overall population	Poor neurological outcome	Good neurological outcome	P value
Patients (n, %)	95 (100)	47 (51)*	46 (49)*	-
Gender (n, %)				0.54
Male	80 (84)	40 (87)	36 (80)	
Female	15 (16)	6 (13)	9 (20)	
Age (years; average ± SD)	36.7±16	42±17.3	32±13.2	0.025
Patients with poor atlas registration (n, %)	31 (33)$	19 (61)	11 (39)	0.06
				
Number of patients studied with the	63 (66)#	27 (43)	36 (57)	-
multivariate analysis (n, %)				

* overall, 2 patients were lost to the follow-up

$ 1 patient with poor atlas registration was lost to the follow-up

# 1 patient with satisfactory atlas registration was lost to the follow-up

n: number, SD: standard deviation

**Fig 1:**
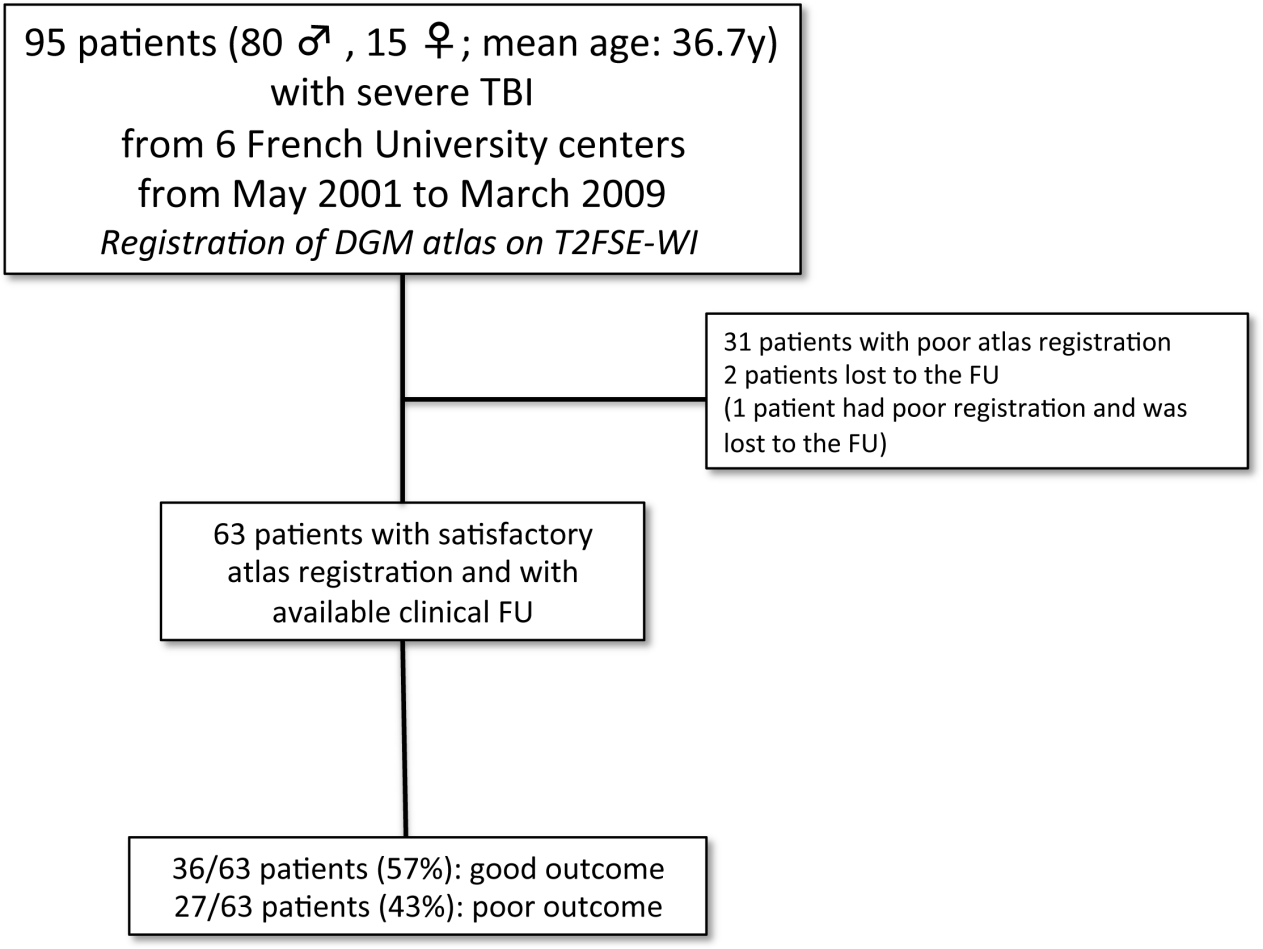
Flow chart summarizing the study design. FU: follow-up.

80 males and 15 females from 15 to 77 years of age (m = 36.7±16) were included in the study. All the patients were hospitalized in the neurosurgical intensive care unit and presented a persistent coma, unexplained by chemical sedation. Neurological prognosis was evaluated on the Glasgow Outcome Scale (GOS) [14] (**Table 2**) at 6 months follow-up. A GOS between 1 and 3 was considered an unfavorable outcome while a GOS of 4 or 5 was considered a favorable outcome. 64 out of the 95 patients (67%) had a satisfactory registration of the DGM atlas. In the overall population, 2 patients were lost to the follow-up; one with a satisfactory DGM atlas registration; one with a poor registration. Thus, a total of 63 patients (66%) had both a satisfactory atlas registration and follow-up available (see the recruitment flow chart, **Fig 1**).

**Table 2:** Glasgow outcome scale (GOS) (14). GOS from 1 to 3 is considered as a poor clinical outcome. GOS of 4 or 5 is considered as a good clinical outcome.

1. Death	Severe injury or death without recovery of consciousness
2. Persistent vegetative state	Severe damage with prolonged state of unresponsiveness and a lack of higher mental functions
3. Severe disability	Severe injury with permanent need for help with daily living
4. Moderate disability	No need for assistance in everyday life, employment is possible but may require special equipment.
5. Low disability	Light damage with minor neurological and psychological deficits.

### Imaging protocol

MRI acquisitions were performed with an average delay of 21±9 days (range: 7–45) from the traumatic insult. The T2 FSE sequence was acquired as part of a multimodal imaging protocol described in a previous paper [8]. T2 FSE sequence parameters in the different institutions where the MRIs were acquired are detailed in **S1 File**.

### Deep grey matter structures

**Fig 2:**
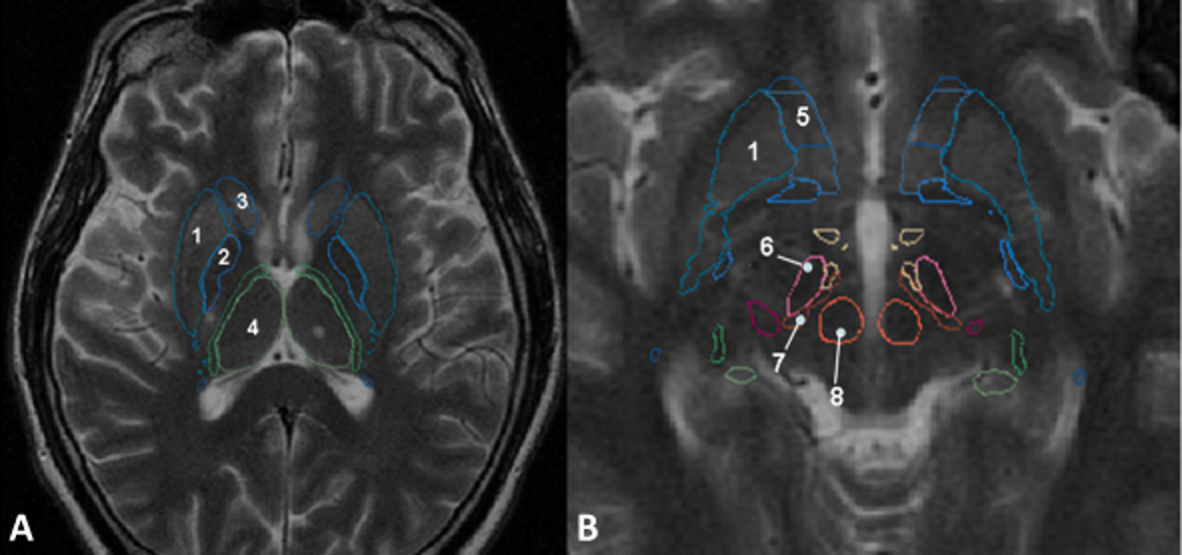
**A**. Registration of the DGM nuclei atlas on axial T2 FSE slice passing through the foramen of Monro. 1. Putamen; 2. *Globus pallidum*; 3. Caudate nucleus head; 4. Thalamus. **B**. Registration of the DGM on axial T2 FSE slice passing through the mesencephalon. 5. Nucleus accumbens; 6. Sub-thalamic nucleus; 7. Zona incerta; 8. Red nucleus.

Our study focused on the following deep grey nuclei: CN, thalamus, putamen, GP, NA, SN, STN, RN and ZI (**Fig 2**). The degree of involvement of the smaller nuclei within the cerebral peduncles (CP), like the pediculopontine nucleus, the *pontis oralis* nucleus or the parabrachial nucleus, which are not yet registered by the DGM nuclei atlas, was indirectly estimated by evaluation of lesions’ volume in CP by means of the semi-quantitative scale described bellow.

### Lesion scoring

**Fig 3:**
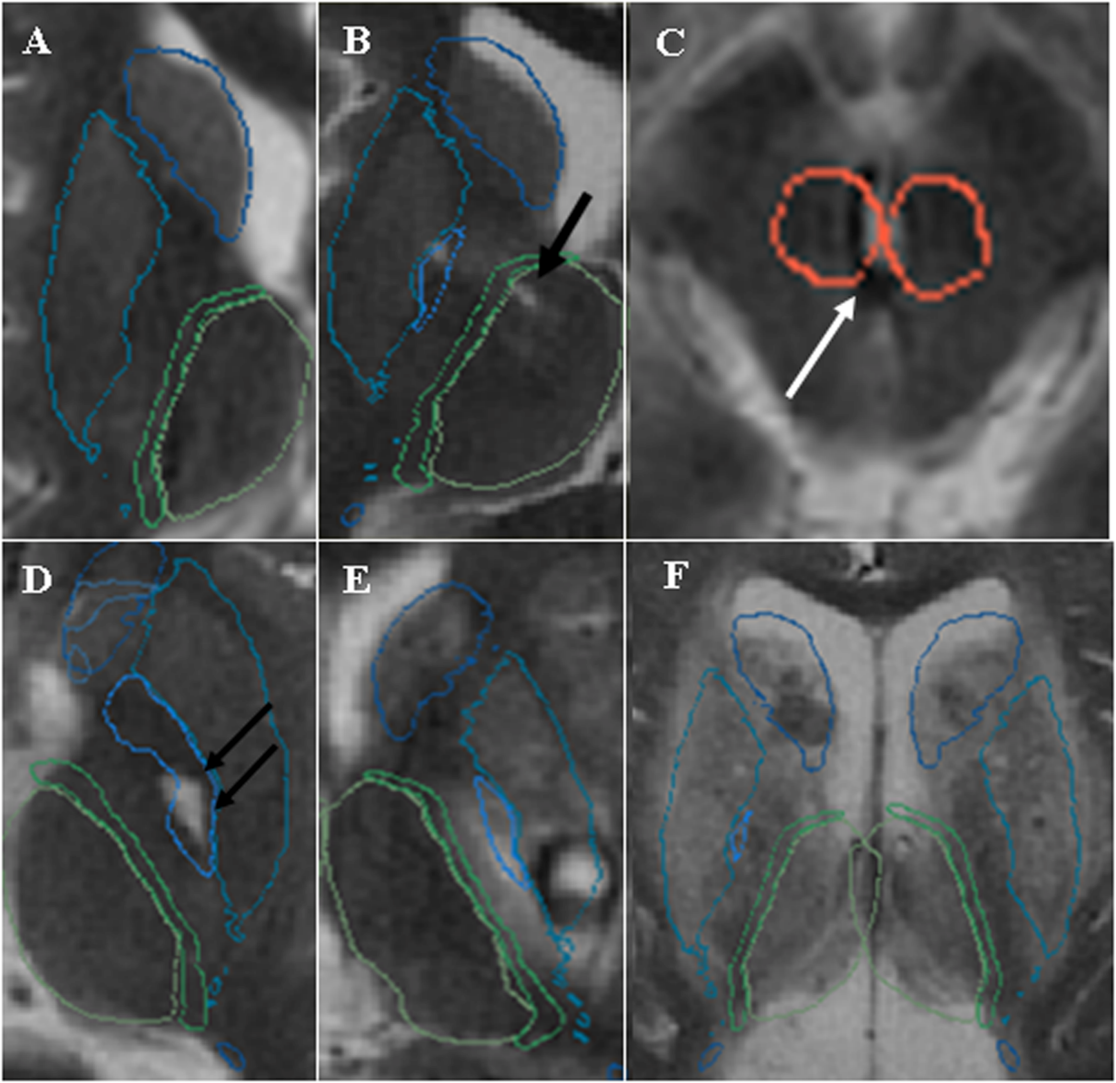
DGML semi-quantitative scale. **A**. No lesion; **B**. Punctuate lesion; **C**. Lesion involving less than 1/3^rd^ of the nucleus. **D**. Lesion involving between 1/3^rd^ and 2/3^rd^ of the nucleus. **E**. Lesion of more than 2/3^rd^ of the nucleus. **F**. Total involvement of the nucleus.

The scoring of the deep gray matter lesions was based on a semi-quantitative scale as follows: 0 = absence of any lesion; 1 = a punctate lesion; 2 = lesion occupying less than 1/3^rd^ of the volume of the nucleus; 3 = lesion occupying between 1/3^rd^ and 2/3^rd^ of the volume; 4 = lesion occupying more than 2/3^rd^ but not the entire nucleus; 5 = involvement of the entire nucleus (**Fig 3**).

If more than one lesion involved one nucleus, the extend of the different lesions involving the same nucleus were added in order to obtain the global traumatic lesions’ burden for a given nucleus.

The volume of CP traumatic lesions was used as a surrogate marker of lesions involving the CP smaller nuclei. The above-mentioned score was used to evaluate the CP lesions’ burden.

The average number of DGML per patient was also evaluated.

All images were independently reviewed by 2 neuroradiologists (FC and DG) with respective experience of 6 and 14 years of experience in neuroradiology.

### Use of a 3D deformable basal ganglia atlas

In order to improve the accuracy of the evaluation of the DGML’ burden, a DGM nuclei atlas was employed in this research [15]. Briefly, this atlas consists of a canonical DGM nuclei template that was derived by correlating histology slices with MRI. For any given patient, this canonical template is mapped on patient specific images by applying an inter-patient, MRI to MRI registration using a non-rigid mapping to account for individual variability in the size and shape of the DGM nuclei. For details on the 3D-deformable DGM nuclei atlas, see **S2 File**.

### Assessment of registration quality

**Fig 4:**
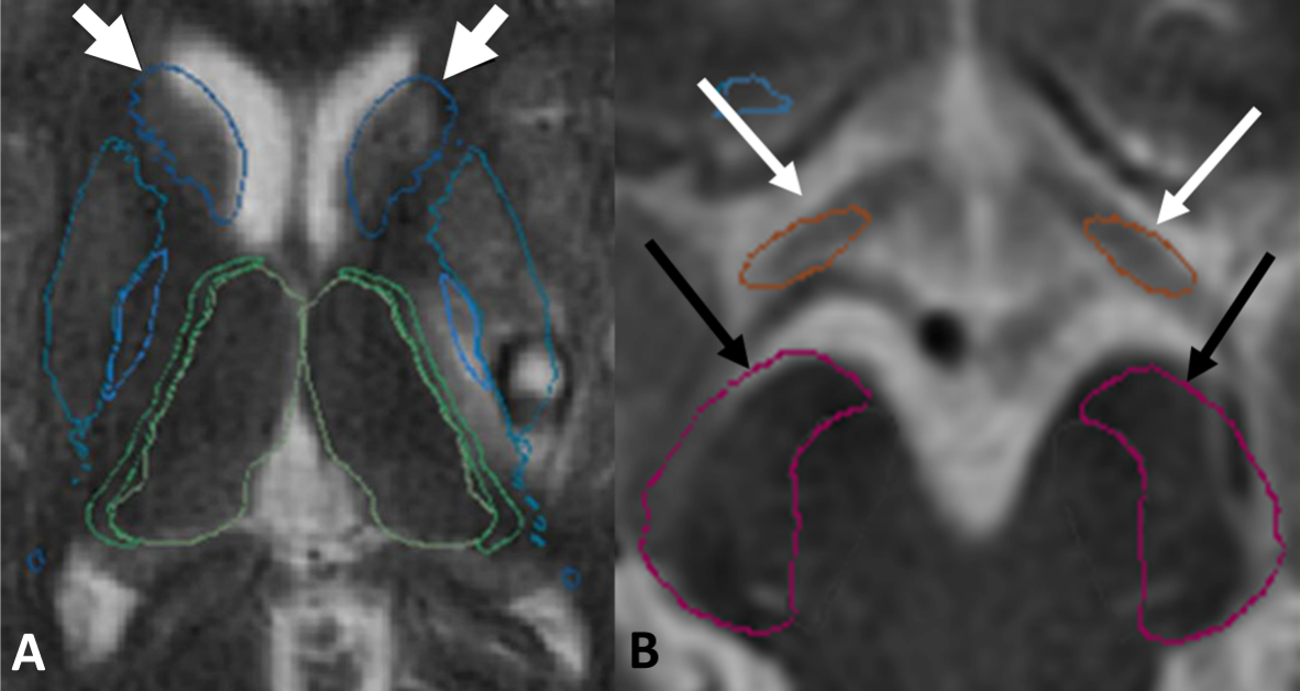
Example of satisfactory registration of the boundaries of the caudate nuclei heads (**A**, white arrows), of the optic tracts (**B**, white arrows) and of the cerebral peduncles (**B**, black arrows).

The quality of the registration of the DGM nuclei atlas on to the T2 FSE images was evaluated qualitatively and quantitatively. For qualitative assessment, the concordance of the following boundaries was checked: medial boundaries of CN heads and thalami (**Fig 4A**); boundaries of CP and optic tracts (OT) (**Fig 4B**). Accurate registration of the boundaries of CN heads and thalami indicated correct registration of the deep gray matter of the cerebral hemispheres while satisfactory registration of CP and OT indicated accurate registration of the brain stem nuclei.

For quantitative assessment, a scale was elaborated to classify the fidelity of registration. For CN heads and thalami, we assigned a score of 0 if less than 10% of the volume of the registered nucleus was bulging into the adjacent ventricle (lateral ventricle for caudate heads, third ventricle for thalami). Scores of 1 and 2 were assigned for an overlap of 10–25% and 25% and more, respectively. For CP and OT, the following scale was used: 0 = no error; 1 = mild error; and, 2 = marked error. Only the patients presenting a correct registration for both the CN heads/thalami (grade 0) and for CP/OT (grade 0) were used for the final analysis of the DGML.

### Statistical analysis

Sensitivity, specificity, positive predictive value (PPV), negative predictive value (NPV), odds ratios (OR), Chi^2^ and Fisher’s exact tests, as well as the Cohen *kappa*-weighted test for the lesion scoring’s inter-rater agreement, were evaluated with the MedCalc software (MedCalc Software version 9.3.2.0, Mariakerke, Belgium).

Multivariate analysis was performed using a Support Vector Machine (SVM) with linear nucleus [16]. Details about SVM are described in **S3 File**.

The multivariate analysis (SVM) was performed on 63 out of 95 enrolled patients (31 patients were excluded due to atlas misregistration; 2 patients were lost on the follow-up; one patient both had misregistration and was lost on the follow-up). The aim of this statistical analysis was to evaluate the weight of lesions of each nucleus of the DGM on the patients’ neurological outcome. Thus, 20 variables (right + left DGM lesions) were used for the multivariate analysis. We also performed the same multivariate analysis using the age as an additional variable (21 variables in total) in order to see if this variable would increase the ability of the classifier to predict good or poor clinical outcome.

The same analysis was performed using the DGML scoring system without the aid of the deformable atlas.

**Fig 5:**
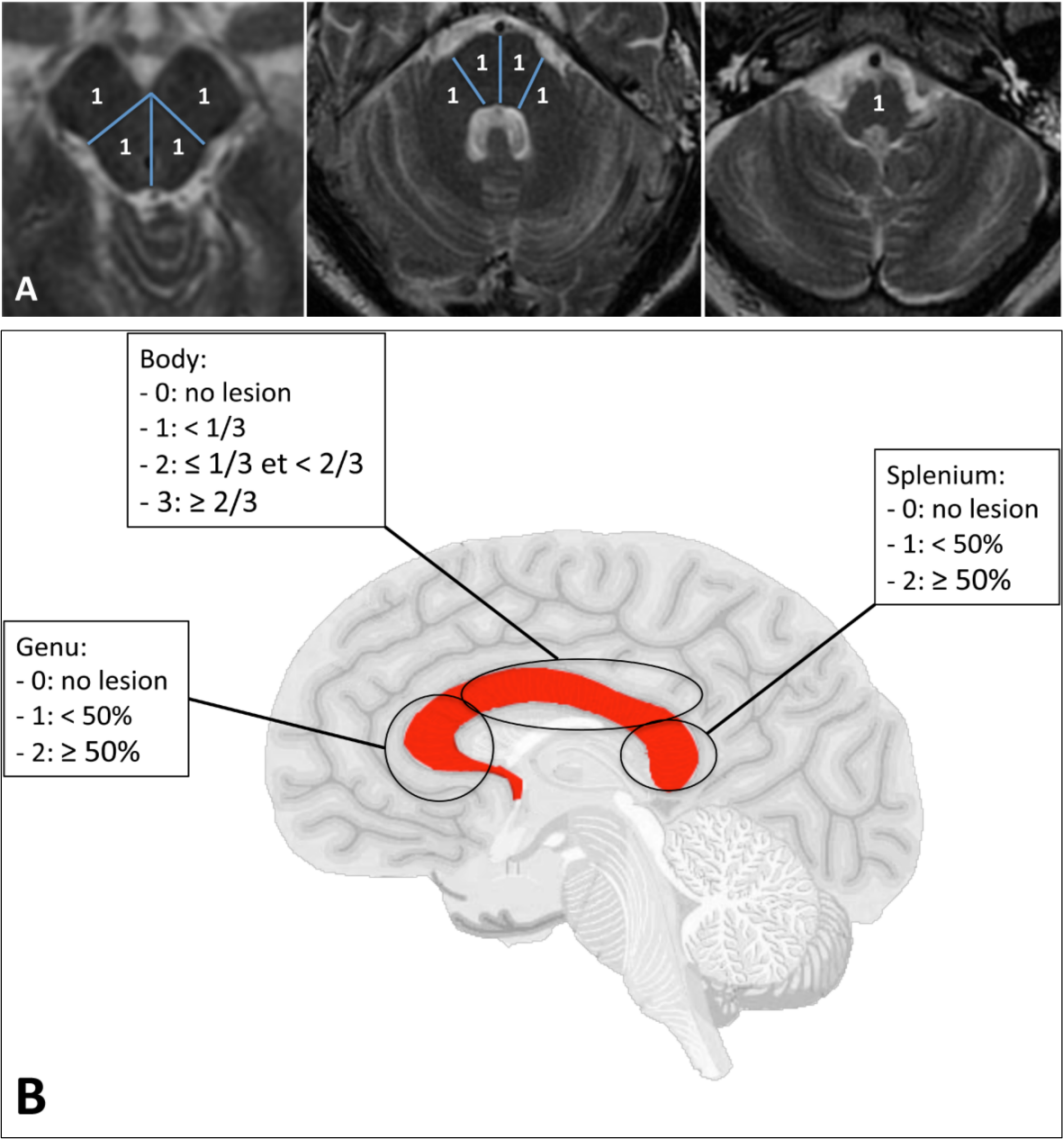
Grading scale combining the evaluation of the lesions’ burden of the brain stem (**A, B** and **C**) and of the corpus callosum (**D**). For the brain stem, the mesencephalon was divided into 4 regions (**A**), the pons into 4 regions as well (**B**) and the medulla oblongata corresponded to one region (**C**). For each lesion involving one region, one point was added (maximum total points = 9). **D**. Drawing of the brain with the corpus callosum colored in red (lateral view). The corpus callosum was divided into 3 areas: the genu, the body and the splenium. No lesion was graded 0, lesion involving < 50% of the genu: 1 point and ≥ 50%: 2 points; lesion involving less than 1/3 of the body: 1 point, ≥ 1/3 and < 2/3: 2 points, and ≥ 2/3: 3 points; lesion involving less than 50% of the splenium: 1 point and ≥ 50%: 2 points. The maximum total points for the corpus callosum was 7 (**D**). By combining the 2 scores, the brain-stem/corpus callosum (BS-CC) score was obtained (maximum points = 16).

Finally, we compared the performances of our grading scale for DGML’ evaluation with a semi-quantitative grading scale combining the evaluation of brain stem and corpus callosum post-traumatic lesion burden (**Fig 5**). Comparison between the areas under the curve (AUC) from the receiver operating characteristic (ROC) curves of the two grading scales was performed using the DeLong's approach [17]. The DeLong's approach is a nonparametric method for computing the AUC that does not need the normality assumption. With this method, the value of AUC is obtained by summing the area of the trapezoids that are formed below the connected points making up the ROC curve.

## Results

### Registration fidelity

**Fig 6:**
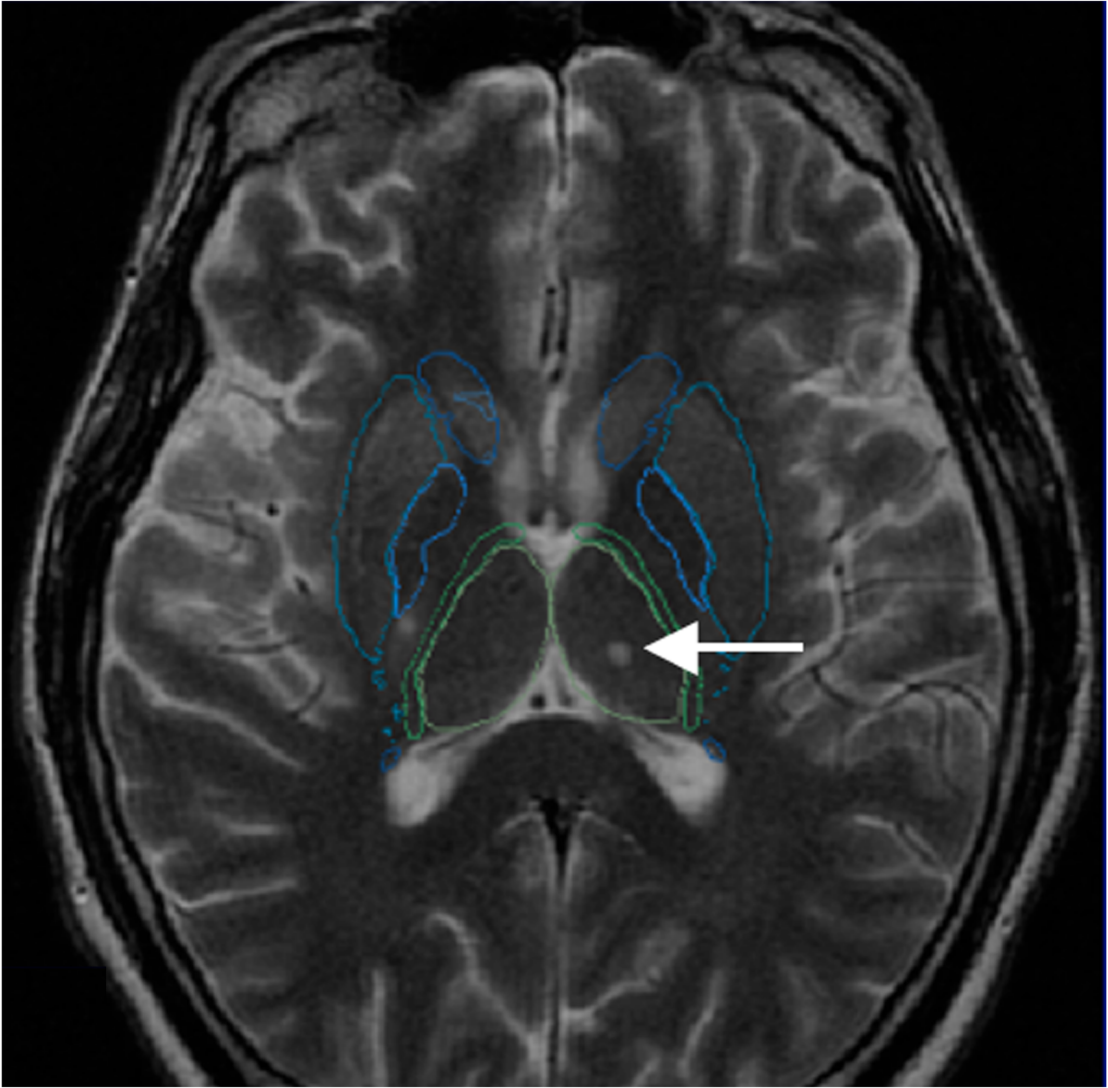
Example of a satisfactory registration of the DGM on a T2 FSE axial slice passing through the thalami (delineated in green) and striatum (delineated in blue). Note the punctuate lesion involving the left thalamus (arrow).

Satisfactory registration of DGM nuclei atlas on T2 FSE images (**Fig 6**) was obtained in 67% of the cases (64/95). Hydrocephalus was present in 9 (56%) of these 16 cases of major misregistration and was statistically associated with a higher rate of major misregistration (Fisher’s exact test: P = 0.000001, OR = 57.2; CI 95% [10.3; 317.8]). Midline shift due to mass effect was also statistically associated with major misregistration (Fisher’s exact test: P = 0.006, OR = 7.8; CI 95% [1.95; 31.4]). Poor registration of the boundaries of the caudate heads was seen in all the cases of misregistrations (31/31); thalami and OT/CP were misregistered in 65% (20/31) and 58% (18/31) of cases, respectively.

### Patients’ outcome

Two patients were lost to the follow-up. In the overall population, 47 (51%) had an unfavorable outcome whereas 46 (49%) had a favorable outcome. Interestingly, patients with favorable outcome were younger than those with poor outcome (32±13.2 vs 42±17.3 years, P = 0.025). On the contrary, sex did not have any influence on the neurological outcome (P = 0.54).

At 6 months, among the 63 patients who had both a satisfactory registration and an available clinical follow-up, 36 (57%) had a favorable (GOS 4–5) outcome whereas 27/63 patients (43%) had an unfavorable (GOS 1–3) outcome.

### Inter-rater agreement for lesions scoring

Interobserver *kappa* test performed to evaluate the agreement between the 2 observers for the DGML scoring was good with a kappa index of 0.71. Only 4% of the studied nuclei presented a scoring gap superior to 1. These were almost always seen in the smallest structures (ZI, SN, STN). These differences were settled by consensus.

### Number and location of DGM lesions

Out of the 64 patients with a good registration of the 3D atlas, 47 (73%) presented at least a lesion in one nucleus. Among these, 139 DGML were counted, with 2.2 DGM lesions on average per patient. DGM regions most frequently affected were: putamen (26%), globus pallidum (16.5%) and thalamus (15%) (**Table 3**). Interestingly, neither age nor sex had any influence on the DGML burden (corresponding to the addition of lesion scoring of each nucleus) (P = 0.91 and 0.27, respectively).

**Table 3:** Characteristics of the deep grey matter lesions in our population.

Characteristics	N (%)
Total Nb of DGML	139
Patients with bilateral lesions	19 (30)
Patients without DGML	18 (28)
**Repartition of DGML**	
Putamen	36 (26)
Globus pallidus	23 (16.5)
Thalamus	21 (15)
Caudate nucleus	20 (14)
SN	18 (13)
STN	6 (4)
ZI	5 (3.5)
Accumbens nucleus	5 (3.5)
RN	5 (3.5)

Percentages are given in brackets

nb: number, DGML: deep grey matter lesions, ZI: zona incerta, SG: substancia nigra, STN: subthalamic nucleus, RN: red nucleus

### Correlation between DGML and neurological outcome

Patients without DGML had a favorable neurological outcome in 89% of cases (16/18) (P = 0.0016, Fisher’s exact test). OR for favorable outcome in patients without DGML was 10 (CI 95% [2.1; 48.7]. Sensitivity, specificity, PPV, NPV for favorable outcome in patients without DGML were 89%, 44.5%, 39% and 91%, respectively. Bilateral lesions were seen in 20 patients. In 16/20 (80%) cases, patients with one or more bilateral DGML had an unfavorable outcome at 6 months (Fisher’s exact test, P = 0.00008, OR = 11.6, CI 95%[3.2; 42.4]). Sensitivity, specificity, PPV and NVP for unfavorable outcome in patients with bilateral lesions were 59%, 89%, 80% and 74.4%, respectively.

The SVM analysis was performed to separate the group “favorable outcome” from the group “unfavorable outcome”. Twenty variables were available, corresponding to the lesion scores of left and right caudate nucleus, putamen, thalami, globus pallidum, accumbens nuclei, subthalamic nuclei, red nuclei, zona incerta, subtansia nigra and cerebral peduncles. The learning set of the multivariate analysis showed that 96.9% of patients with unfavorable outcome and 96.8% of patients with favorable clinical outcome were correctly classified by the discriminant function, respectively. The Jacknife cross-validation method found 90.5% of correct classification in the “unfavorable outcome” group and 81% in the “favorable outcome” group (**Table 4**). When the multivariate analysis was performed with “age” as an additional variable, the classification accuracy was not significantly increased (91.3% for unfavorable neurological outcome).

**Table 4:** Cross validation (Jacknife test). In each validation test, we used data from all but one subject (S -1 of the S subjects) to train the classifier.

Predicted/Initial	Good	Poor	
**Good**	34 (94.4%)	8 (29.6%)	Good predictive value: 81%
**Poor**	2 (5.6%)	19 (70.4%)	Poor predictive value: 90.5%

Comparison of the AUC from ROC curves of the DGML scoring with and without the aid of the DGM nuclei atlas showed that the results obtained with the atlas were more accurate to predict poor neurological outcome (0.868, 95% CI [0.763–0.974] with the atlas vs 0.774, 95% CI [0.63–0.918] without the atlas). However, the difference was not statistically significant (Z = 1.6065, P = 0.1082).

**Fig 7:**
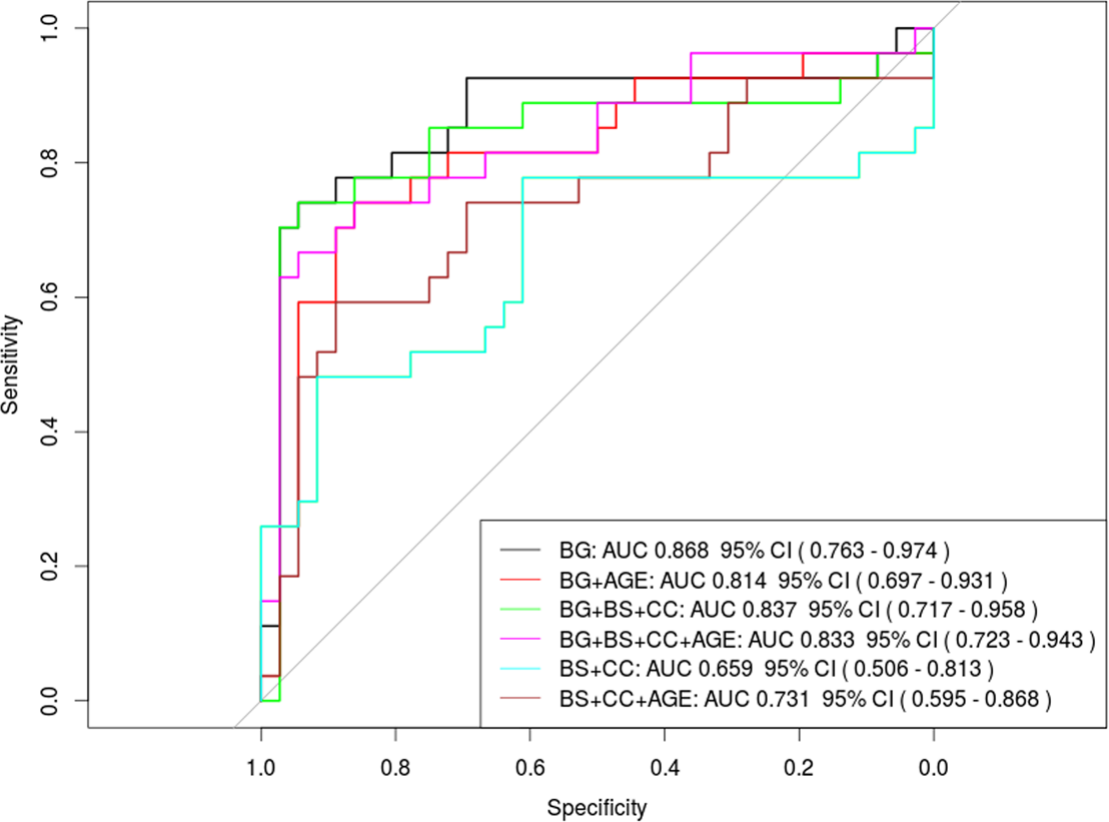
Receiver operating characteristic (ROC) curves and area under the curve (AUCs) for the different grading scales used in this studies, alone or in combination, with or without the factor “age” taken into account. Note that the AUC of the ROC curve of the DGM nuclei grading scale was higher than the one of the other scores, alone or combined, with or without the age.

Comparison of the AUC from ROC curve of our DGML grading scale versus the ones of brain stem and corpus callosum grading scale showed that the DGML grading scale was more accurate in predicting a poor neurological outcome (Z = 2.695, P = 0.007). Interestingly, the combination of DGML, brain stem and corpus callosum grading scales did not improve the accuracy in predicting a poor neurological outcome (**Fig 7**).

## Discussion

Some predictive factors for unfavorable neurological outcome in severe TBI have already been described, in particular the 2 weeks’ GCS [18], the shear-related white matter lesions on MRI [19] or the white matter lesions depicted on multimodal imaging (DTI, MR spectroscopy) [8, 20]. However, they still cannot correctly predict the individual neurological outcome for a large number of patients. In the present study, we chose to investigate the DGML because we hypothesized, according to our clinical experience, that few strategic small lesions affecting the DGM may be associated with a worse outcome than one voluminous brain lesion.

### DGML count

After atlas registration, 71% of the patients had DGML. This rate is higher than previously reported in the literature, in which it ranges from 19 to 46% [21–24].

In the literature, only few studies analyzed individually DGML in TBI. Kampfl et al. [22], in a prospective study on 80 patients with severe TBI, found that traumatic lesions involved the thalamus in 46% of cases; lenticular nuclei in 38% and caudate nuclei in 10%. Other series focused on the rate of thalamic traumatic lesions; especially neuropathological studies [25, 26], in which this rate varies from 60 to 80% in patients who died or remained in vegetative state.

### Neurological outcome and DGML

Our work underlines the correlation between unfavorable neurological outcome and DGML. The absence of DGM lesion on T2 FSE after atlas fusion was strongly predictive of favorable neurological outcome (89%, OR = 10). On the contrary, bilateral lesions of DGM nuclei were associated with an 80% rate of unfavorable outcome (OR = 11.6). Additionally, on the basis of the scoring of the DGM nuclei lesions using the atlas, the discriminative function allowed an accurate classification of patients with unfavorable outcome at 6 months with a rate of 90.5%.

According to the data in the literature, DGML are the third most common intracranial parenchymal lesions in severe TBI, after corpus callosum and brain stem [22], which are often integrated in the large group of diffuse axonal injuries (DAI) [27].

Few series have studied the relationship between DGML and neurological outcome in severe TBI [22]. One retrospective MRI study [23] on 15 patients (children with severe TBI) showed a rate of pejorative neurological outcome of 95% for patients with DGM lesions associated with brain stem involvement. Others series have demonstrated the strong implication of thalamic lesions in unfavorable neurological outcome [26, 28–30]. However, in our study, the multivariate analysis did not show that lesion of any given nucleus was by itself predictive of a poor outcome.

Additionally, in our study, the evaluation of DGML helped to predict more accurately a poor neurological outcome than other grading schemes involving brain regions known to be frequently involved in severe TBI like the corpus callosum and the brain stem [22, 31].

### Interest of using a 3D deformable atlas

The originality of this work was to present a precise anatomic analysis of DGM lesions in severe TBI, using a 3D deformable atlas. The use of the 3D atlas was motivated by the fact that DGM is difficult to see on clinical MRI. While T2 FSE is one of the most adapted sequences for exploration of severe TBI lesions [32, 33], it cannot precisely determine the involvement of the DGM, due to the small size of some structures and their poor contrast with the rest of the parenchyma. Due to deformations of brain parenchyma secondary to TBI and the interindividual variation in normal anatomy, a deformable atlas was necessary to precisely localize the lesions. Yet, commonly used DGM nuclei atlases, like the Schaltenbrand and Wahren atlas [34], do not present elastic deformable characteristic and may give erroneous localization of small deep gray structures. In our study, there was a tendency for better performance in predicting poor neurological outcome using the atlas than without; however, this difference was not statistically significant (p = 0.1082). This may be explained by a lack of statistical power due to a too small population sample size (63 patients).

### Limitations

Our study presents some limitations. The volume of the population of our series remains small with respect to the number of variables taken into account in the multivariate analysis. Some of the DGM nuclei were rarely involved (like ZI, which presented lesions in only 4% of the cases). This fact does not allow precise evaluation of their implication in unfavorable outcome.

In our study, the DGM nuclei atlas was, most of the time (67%), successfully registered on the T2 FSE. Fusion was totally non-interpretable, due to poor registration, in only 16/95 cases (17%). Moreover, the criteria used to consider a registration as accurate were very restrictive, leading to exclude 31 patients (33%) from the analysis. Most of the misregistrations were due to an overhanging of the CN in the lateral ventricles and were observed in cases of hydrocephalus and/or midline shift. Methodological developments on the atlas used are underway to minimize these errors, especially in case of ventricular dilatation.

Registration of the DGM nuclei atlas on susceptibility-weighted imaging (SWI) or 3D Fluid attenuation inversion recovery (FLAIR) volumes may have improved the evaluation of DGML’ burden. However, these sequences were not available for clinical use in all centres participating in the study during the inclusion period. Additionally, even if T2 GE or SWI are highly sensitive sequences for the depiction of post-traumatic lesions, they only display haemorrhagic ones. It should also be mentioned that the hypo-intense signals related to the hemoglobin degradation products seen on these sequences are artefacts. They thus cannot be measured to evaluate the extend of the lesion burden.

Finally, some old ischemic lesions of the deep grey matter may have been wrongly interpreted on T2 FSE as post-traumatic lesions, leading to an overestimation of the lesions’ burden. However, our population was young (mean age = 36.7 y), and the probability of such ischemic lesions in young patients is low.

## Conclusion

DGML were frequently observed in our study population with severe TBI. Multivariate analysis performed on the basis of DGML scores may predict poor neurological outcome at 6 months’ follow-up in severe TBI, with a 90.5% positive predictive value. These results need further studies in order to determine if an algorithm combining DGM lesions evaluation with other clinical/imaging/functional data may increase the accuracy of the prediction of outcome in severe TBI.

## Supporting information

S1 File Details of the T2 FSE acquisitions in each center involved in the study. Nb: number, FOV: field of view, ET: echo time, RT: repetition time.
(DOCX)

S2 File Description of the atlas used in the study. (DOCX)

S3 File Details about support vector machine. (DOCX)
