# Supplementary material for: Lesions in deep gray nuclei after severe traumatic brain injury predict neurologic outcome
Source: PLoS One. 2017 Nov 2;12(11):e0186641. doi: 10.1371/journal.pone.0186641 (PMC5667824; doi:10.1371/journal.pone.0186641)
Supplement: S1 File — Nb: number, FOV: field of view, ET: echo time, RT: repetition time. (DOCX) [file pone.0186641.s001.docx]

***Center MRI field MRI Matrix ET RT Slice thickness Interslice***

***Nb strength (mm) gap (mm)***

1. 1.5T GE Signa Excite 256x256 104.8 3340 3 0

3T GE Signa Hdx 512x256 92.2 3420 3 0

1. 1.5T Siemens Sonata 256X192 129 5680 3 0
2. 1.5T Symphony Tim 256x256 103 5580 3 0
3. 1.5T GE Signa Hdx 256x256 92 4900 3 0

1. 1.5T Philips Intera 256x203 92 4860 3 0
2. 1.5T GE Signa Hdx 256x256 91 7000 3 0

***Supplemental material # 1***

Details of the T2 FSE acquisitions in each center involved in the study.

Nb: number, FOV: field of view, ET: echo time, RT: repetition time
