# Supplementary material for: Lesions in deep gray nuclei after severe traumatic brain injury predict neurologic outcome
Source: PLoS One. 2017 Nov 2;12(11):e0186641. doi: 10.1371/journal.pone.0186641 (PMC5667824; doi:10.1371/journal.pone.0186641)
Supplement: S2 File — (DOCX) [file pone.0186641.s002.docx]

**Supplemental material # 2:**

***Description of the atlas used in the study***

The 3D-deformable basal ganglia atlas was generated from autopsy material of a donor with no neurological or psychiatric disease. A detailed MR scan was performed on this brain using a protocol that included the following two specific sequences: (a) T1-WI (3DIR-FSPGR, TR = 11 ms, TE = 2 ms, TI = 600 ms, flip angle = 10°, NEX = 1, slice thickness = 1.3 mm, interslice gap = 0) and (b) T2 spin echo (TR = 2000 ms, TE = 90 ms, NEX = 1, slice thickness = 2 mm, interslice gap = 1 mm). After extraction of the brain from the skull, its hemispheres were separated and put into formol solution. “En bloc” coronal sections were performed, followed by a cryosection with a microtome (thickness = 70 μm) of the left hemisphere fixed in formol, and then frozen. After histological and immunohistochemical colorations, a delineation of the BG and thalamus, and their functional territories was performed.
Fusion between images obtained from the histological atlas and MR images of the post-mortem brain enabled reconstruction of surface models of BG.

A deformation strategy was developed to morph the histological 3D atlas on to T1 and T2-weighted MR images. This was done in two steps. First, the histological atlas was mapped on the MR images using a global registration via a rigid transformation. This global registration accomplished coarse matching between the histologic and MR data sets. This was followed by fine registration using a non-rigid mapping based on homology between regions of interest including BG on MR images of a given patient and MR images of the atlas.
