# Supplementary material for: Lesions in deep gray nuclei after severe traumatic brain injury predict neurologic outcome
Source: PLoS One. 2017 Nov 2;12(11):e0186641. doi: 10.1371/journal.pone.0186641 (PMC5667824; doi:10.1371/journal.pone.0186641)
Supplement: S3 File — (DOCX) [file pone.0186641.s003.docx]

**Supplemental material # 3**

***Details about support vector machine***

Currently, support vector machine (SVM) is considered as the standard of classification methods in pattern recognition learning machine. SVM performs classification by constructing an *N*-dimensional hyperplane that optimally separates the data into two categories without hypothesis on variable distributions. The non-linear mapping into a high dimensional space is affected by a kernel which could be a polynomial function, Gaussian, radial basic function, or a multi-layer perceptron. The aim of the multivariate analysis was to find a discriminative function to separate the group “favorable neurological outcome” (GOS = 4 or 5) from the group “unfavorable neurological outcome” (GOS from 0 to 3). We evaluated the performance of the classifier using the leave-one-subject-out cross validation test, also known as the Jacknife test. In this test, we used data from all but one subject to train the classifier. Subsequently, the class assignment of the remaining subject, which was so far not seen by the algorithm, was calculated. If there are S data points, this procedure was repeated S times, each time leaving out a different subject. A nonparametric approach, such as permutation tests, can estimate empirically the distribution of the statistic under a null hypothesis. Nonparametric tests have been previously applied to fMRI data analysis [9]. By permuting the class labels 100 times randomly and training the SVM with this permutation of labels, we estimated a probability distribution for each variable v_i_ under the null hypothesis of no relationship between the class labels and the global structure of the data. Based on this probability distribution and its mean (m) and its standard deviation (s), it is possible to evaluate the normalized weight of each variable v_i_ by testing (v_i_ – m)/s.
